# *Toxoplasma gondii*-induced ferroptosis contributes to acute lung injury in mice

**DOI:** 10.1186/s13071-025-07207-x

**Published:** 2025-12-28

**Authors:** Xiaodan Yuan, Zhenzhen Liu, Yeting Ma, Feixue Liu, Penglin Bao, Boya Du, Xu Zhang, Pengtao Gong, Nan Zhang, Jianhua Li, Xin Li, Xiaocen Wang

**Affiliations:** State Key Laboratory for Zoonotic Diseases, Key Laboratory for Zoonosis Research of the Ministry of Education, Institute of Zoonosis, and College of Veterinary Medicine, Jilin University, Changchun, 130062 PR China

**Keywords:** *Toxoplasma gondii*, Lung injury, Ferroptosis, Iron, Antioxidant, Deferiprone

## Abstract

**Background:**

*Toxoplasma gondii* (*T. gondii*) is an important apicomplexan parasite that causes zoonotic toxoplasmosis in humans and animals. Acute *T. gondii* infection leads to systemic immunopathology that may manifest as lung injury or pulmonary embolism. Ferroptosis is an iron-dependent regulated cell death driven by lethal lipid hydroperoxide accumulation. Emerging evidence implicates ferroptosis in infection-related tissue damage; however, the role of ferroptosis in *T. gondii*-induced lung injury remains to be explored.

**Methods:**

Mice were infected with *T. gondii* to establish a lung injury model. The body weight changes, survival rate, inflammatory cytokines, lung histopathology, and parasite burden were assessed. The key ferroptosis-related indicators involved in antioxidant, iron metabolism, and lipid metabolism pathways were analyzed in lung tissues using techniques such as transmission electron microscopy, western blotting, and immunohistochemistry. Deferiprone (DFP), an oral iron chelator that can inhibit ferroptosis, was used to investigate the potential role of ferroptosis in *T. gondii* lung injury.

**Results:**

*T. gondii* infection induced lung injury in mice with thickening of alveolar septa and hemorrhage in alveolar spaces, accompanied by iron deposition. Crucially, *T. gondii* triggered ferroptosis in lung tissues of mice, evidenced by MDA elevation, GSH depletion, total iron and Fe^2+^ overload, and mitochondrial cristae loss. Furthermore, iron metabolism pathways were disordered while antioxidant pathways were suppressed. DFP treatment reversed ferroptosis alterations, decreased inflammatory cytokines, attenuated pathological changes, reduced *T. gondii* burden, and prolonged survival of the infected mice.

**Conclusions:**

Our findings revealed that *T. gondii* infection triggered ferroptosis by compromising dysregulated iron metabolism and antioxidant defenses, playing a key role in *T. gondii*-induced lung injury. DFP exhibited a promising therapy effect for toxoplasmosis.

**Graphical Abstract:**

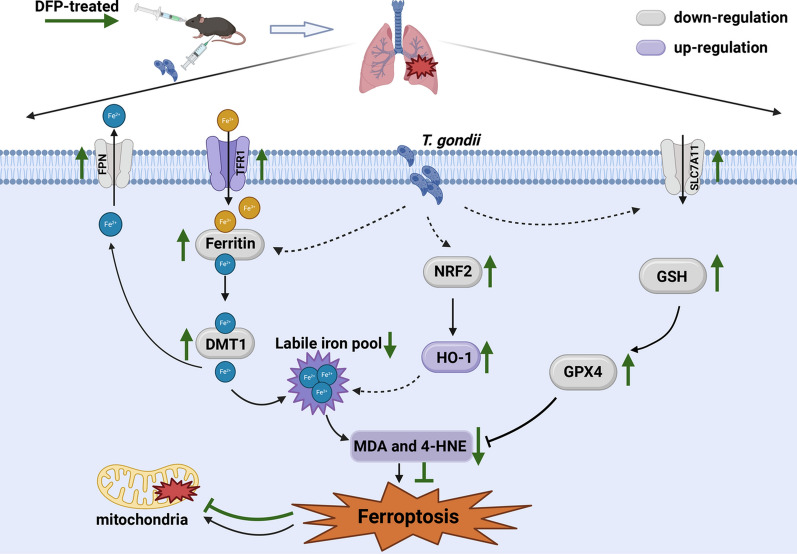

**Supplementary Information:**

The online version contains supplementary material available at 10.1186/s13071-025-07207-x.

## Background

*Toxoplasma gondii* is a globally distributed apicomplexan parasite that infects nearly all warm-blooded animals, chronically infecting more than 30% of the human population [1–3]. While typically asymptomatic in immunocompetent individuals, primary infection during pregnancy can lead to miscarriage, stillbirth, or congenital defects. Acute infection is characterized by rapid replication of tachyzoites and dissemination to multiple tissues, such as brain and skeletal muscle, usually followed by the establishment of chronic infection, which is characterized by the formation of tissue cysts [4–6]. Under immunosuppressive conditions, bradyzoites may reactivate into rapidly replicating tachyzoites, causing encephalitis, pneumonia, chorioretinitis, or myocarditis [7–10]. In immunocompromised hosts, *T. gondii* infection may evolve into persistent pulmonary infiltrates and progressive respiratory insufficiency, frequently culminating in misdiagnosis and heightened mortality [11, 12]. Researches have shown that *T. gondii*-induced lung injury results from a combination of factors including tachyzoite proliferation-mediated cell lysis, dysregulated host immune responses, particularly excessive proinflammatory cytokine production, and associated oxidative stress [12–14]. However, the underlying pathogenic mechanisms of lung injury caused by *T. gondii* remain poorly understood.

Ferroptosis is driven by the accumulation of reactive oxygen species (ROS) and iron-mediated oxidative stress, ultimately leading to cellular membrane damage and organ dysfunction [15, 16]. This process is characterized by mitochondrial shrinkage, lipid ROS buildup, and dysfunction of the glutathione peroxidase 4 (GPX4)–glutathione (GSH/GSSH) antioxidant axis [15–17]. Ferroptosis is primarily modulated with iron metabolism, antioxidant system, lipid metabolism [16], mainly including the accumulation of ROS derived from labile iron (Fe^2+^), which catalyzes the Fenton reaction, generating highly reactive hydroxyl radicals, the system Xc⁻–GSH–GPX4 pathway [13, 16], the ferroptosis inhibitory protein 1–CoQ10 pathway, the peroxidation of polyunsaturated fatty acids in cellular membranes, and the GTP cyclohydrolase-1 and tetrahydrobiopterin/dihydrobiopterin [17].

Recent studies highlight an important link between ferroptosis and various diseases, such as cancers, degenerative diseases, infectious diseases, and autoimmune diseases. Pharmacological inducers of ferroptosis, including erastin, have been shown to suppress tumor growth by selectively promoting ferroptosis, exhibiting significant antitumor activity against various cancer types, such as lung cancer cells, osteosarcoma cells, and breast cancer cells [18–20]. Recently, ferroptosis inhibitors, DFP and desferrioxamine, were used in treating Parkinson’s disease and Alzheimer’s disease, respectively [21–23]. As for infectious diseases, several pathogens may exacerbate tissue injury and inflammation by manipulating ferroptosis. Herpes simplex virus 1-induced ferroptosis contributes to viral encephalitis [24]. *Mycobacterium tuberculosis* (*M. tuberculosis*) [25] and SARS-CoV-2 [26] induce lung ferroptosis by disrupting iron homeostasis and enhancing oxidative stress, ultimately leading to lung injury. *Clonorchis sinensis* (*C. sinensis*)-induced hepatic ferroptosis exacerbates liver fibrosis [27]. *Plasmodium berghei* (*P. berghei*) could induce ferroptosis, causing liver injury in mice [28]. During *T. gondii* acute and chronic infection, *T. gondii* promotes liver and brain ferroptosis and enhances its proliferation by inhibiting GPX4 [29]. Furthermore, *T. gondii* drives ferroptosis in the retina, brain, spleen, liver, and kidney, manifesting as excessive iron accumulation and ROS generation by hijacking of host iron-lipid metabolism [30–32]. However, whether ferroptosis also affects the lungs during acute toxoplasmosis remains unclear. In addition, ferroptosis can also inhibit Zika virus and *Brucella* survival by eliminating infected cells, thereby contributing to host defense [33, 34]. Thus, targeted modulation of ferroptosis presents a promising strategy for controlling a range of diseases. Nonetheless, its potential role in protecting against lung injury during acute *T. gondii* infection remains to be systematically investigated.

In this study, we aimed to elucidate the contribution of ferroptosis to lung injury during acute *T. gondii* infection and to evaluate whether iron chelation could attenuate lung injury, providing a basis for the development of therapeutic strategies against *T. gondii* infection.

## Methods

### Parasite

*Toxoplasma gondii* (RH strain) tachyzoites were maintained by serial passages in Vero cells cultured in Roswell Park Memorial Institute (RPMI)−1640 medium supplemented with 2% fetal bovine serum (FBS; cat. no. C4001; VivaCell, Shanghai, China), at 37 °C in a humidified 5% CO₂ incubator. Tachyzoites were harvested using 40% (v/v) Percoll (cat. no. P8370; Solarbio, Beijing, China) in phosphate-buffered saline (PBS) by gradient centrifugation as previously described [35].

### Animals and infection experiment

The 6-week-old female C57BL/6 mice were obtained from Liaoning Changsheng Biotechnology Co., Ltd. (Benxi, China). Mice were housed under specific pathogen-free conditions (12 h light/dark cycle, 50–60% humidity, 25 °C) with ad libitum access to sterile food and water. Animals were acclimatized for 1 week before experiments.

For the lung injury model, mice were randomly divided into the normal control (NC) group and the *T. gondii* infection (RH) group (*n* = 10 mice/group). Mice in the RH group were intraperitoneally (i.p.) injected with 1 × 10^3^ tachyzoites. Mice in the NC group received an equal volume of PBS.

To evaluate the effect of ferroptosis on *T. gondii* infection, mice were randomly divided into four groups (*n* = 10 mice per group), namely the RH group, DFP + RH group, NC group, and DFP group. In the DFP group and DFP + RH group, mice were intragastric administrated (i.g.) daily with 100 mg/kg DFP (an inhibitor of ferroptosis; cat. no. HY-B0568; MCE, New Jersey, USA); then in the DFP + RH group mice, were challenged with 1 × 10^3^ tachyzoites i.p. on the second day after the first administration of DFP. In the RH group and NC group, mice were administrated (i.g.) daily with an equal volume of sterile water.

Mice were monitored daily for changes in body weight and survival and either assessed using a humane scoring system (see Additional file 1: Supplementary Table S1). Once the total score reached six, they were considered moribund and humanely euthanized [36]. Mice that were euthanized upon reaching these predefined humane endpoints were included as events in the survival analysis. Detailed information is listed in Additional file 1: Supplementary Tables S2 and S3. At 6 days post infection (dpi), five mice from each group were euthanized for terminal sample collection. Serum samples and lung tissues were harvested and immediately snap-frozen in liquid nitrogen [30, 31].

### Cytokine measurement

Serum concentrations of interleukin (IL)−12, IL-6, interferon (IFN)-γ, and tumor necrosis factor (TNF)-α were quantified using commercial enzyme-linked immunosorbent assay (ELISA) kits (IL-12: cat. no. 88–7120-22; IL-6: cat. no. 88–7064-22; IFN-γ: cat. no. 88–7314-22; TNF-α: cat. no. 88–7324-22; all from Thermo Fisher Scientific, San Diego, CA, USA), according to the manufacturer’s instructions.

### Histopathology examination

The left lung tissues were fixed in 4% paraformaldehyde (cat. no. G1101; Servicebio, Wuhan, China) for more than 24 h, then processed through dehydrated, cleared, and paraffin embedded. Sections (4 μm) were stained with hematoxylin and eosin (H&E) to assess histopathological changes [12]. Iron deposition was analyzed by Prussian blue staining using a commercial kit (cat. no. G1424; Solarbio, Beijing, China) according to the manufacturer’s protocol. Finally, the staining results were observed and captured using an Olympus BX43 microscope (Olympus, Japan) equipped with a digital BioHD-C20 camera (FluoCa, China). Images were acquired at a resolution of 5440 × 3648 pixels. For consistent presentation, white balance was applied uniformly across H&E and Prussian blue staining using Adobe Photoshop 25.3.1 software. No other digital manipulations were used.

### Measurement of malonaldehyde, reduced glutathione, and iron contents

Frozen lung tissues were homogenized using a homogenizer (DHS SV48R; Ningbo, China). For each sample, 50 mg of the resulting homogenate was weighed and further homogenized in 500 μL homogenization buffer according to the manufacturer’s protocols. After centrifugation at 12,000 g for 10 min at 4 °C, the protein concentration in supernatants were detected using a bicinchoninic acid (BCA) protein assay kit (cat. no. C503021; Sangon Biotech, Shanghai, China). Malonaldehyde (MDA) and reduced glutathione (GSH) levels were analyzed using commercial kits (MDA, cat. no. A003-1; GSH, cat. no. A006-2; both from Nanjing Jiancheng Bioengineering Institute, Nanjing, China). For iron contents, both ferrous ion (Fe^2+^) and total iron contents in lung tissues were measured using a Fe^2+^ assay kit (cat. no. BC4355; Solarbio) and a tissue iron assay kit (cat. no. BC5415; Solarbio), respectively. Serum iron concentration was detected by the Serum Ferri Ion Content Assay Kit (cat. no. BC1735; Solarbio).

### Transmission electron microscopy (TEM)

Lung samples were fixed in 2.5% glutaraldehyde (cat. no. G1102; Servicebio) at 4 °C for 2–4 h in the dark. Subsequently, the samples were postfixed with 1% osmium tetroxide at 4 °C for 2 h. After washing, the fixed samples were dehydrated through a graded ethanol series and acetone. The dehydrated samples were then embedded in Epon 812 (cat. no. 90529-77-4; SPI, USA) and polymerized at 60 °C for 48 h. Ultrathin sections (60–80 nm) were cut and double-stained with uranyl acetate-lead citrate [37]. Images were obtained using a TEM system (JEM1400PLUS; NEC Corporation, Japan).

### Western blot analysis

Approximately 25 mg of lung tissue was lysed in 250 μL radioimmunoprecipitation assay buffer (cat. no. P0013B; Beyotime, Shanghai, China), containing 1 mM phenylmethylsulfonyl fluoride (cat. no. MF105; Mei5bio, Beijing, China) and phosphatase inhibitors (cat. no. MF183; Mei5bio), then sonicated. Protein concentrations were quantified using a BCA protein assay kit (cat. no. C503021; Sangon Biotech). Equal amounts (30 μg) of protein were separated by sodium dodecyl sulfate–polyacrylamide gel electrophoresis (SDS–PAGE) and transferred to polyvinylidene fluoride (PVDF) membranes. After blocking in skim milk (5% w/v in phosphate-buffered saline with Tween 20 (PBST)), the membranes were incubated overnight at 4 °C with specific primary antibodies. Separate membranes were incubated with anti-transferrin receptor 1 (TFR1), anti-divalent metal ion transporter 1 (DMT1), anti-ferroportin (FPN), anti-ferritin, anti-nuclear factor erythroid 2-related factor 2 (NRF2), anti-heme oxygenase 1 (HO-1), anti-solute carrier family 7 member 11 (SLC7A11), anti-glutathione peroxidase 4 (GPX4), and anti-β-actin. A horseradish peroxidase (HRP)-conjugated goat anti-rabbit secondary antibody was then used for detection. Chemiluminescent signals were visualized using a digital imaging system (Clinx, Shanghai, China). Complete antibody information is listed in Table 1.

**Table 1:** Detail information of antibodies used in this study

Antibody name	Western blot	IHC	Source	Cat. no.	Supplier
Anti-TFR1 antibody	1:1,000	–	Rabbit	A25900	Abclonal, China
Anti-DMT1 antibody	1:1,000	–	Rabbit	HA723078	Huabio, China
Anti-HO-1 antibody	1:2,000	–		HA721854	
Anti-NRF2 antibody	1:1,000	1:200	Rabbit	ab76026	Abcam, UK
Anti-ACSL4 antibody	1:1,000	–		ab155282	
Anti-KEAP1 antibody	1:1,000	–	Rabbit	HY-P80732	MCE, USA
Anti-4-HNE antibody	1:1,000	1:200		HY-P81208	
Anti-SLC7A11 antibody	1:1,000	–	Rabbit	F3380	Selleck, USA
Anti-GPX4 antibody	1:1,000	1:200		F1580	
Anti-ferritin antibody	1:1,000	1:200		F3878	
Anti-FPN antibody	1:1,000	1:50	Rabbit	26,601-1-AP	Proteintech, China
Anti-β-actin antibody	1:10,000	–		20,536-1-AP	
HRP-goat anti-rabbit recombinant secondary antibody	1:5,000	–		SA00001-2	

### Immunohistochemistry (IHC)

Paraffin-embedded lung sections were deparaffinized, rehydrated, and subjected to antigen retrieval and blocking. Sections were then incubated overnight at 4 °C with primary antibodies against Ferritin, FPN, GPX4, HO-1 or 4-hydroxynonenal (4-HNE), followed by incubation with HRP-conjugated secondary antibodies (detailed information is listed in Table 1). The sections were counterstained with hematoxylin and mounted with neutral resin [38]. Digital images were captured using a light microscope (Olympus BX43, Japan).

### Parasite burden in lungs

Genomic DNA was extracted from a homogenate of the entire right lung using a DNA extraction kit (cat. no. DP304; TIANGEN, Beijing, China), and its concentration and purity were assessed using a Nanodrop 2000 spectrophotometer (Thermo Scientific). A standard curve was generated using tenfold serial dilutions of *T. gondii* genomic DNA, corresponding to a range of 0–1 × 10⁷ tachyzoites, with each dilution run in three replicates [39]. The parasite burden was quantified by quantitative PCR (qPCR) targeting the *T. gondii B1* gene, using genomic DNA (200 ng) and the following primers: forward, 5′–TCCCCTCTGCTGGCGAAAAGT–3′ and reverse, 5′–AGCGTTCGTGGTCAACTATCGATTG–3′ [40]. Each 20-μL reaction contained a 2× SYBR Green Master Mix (cat. no. G891; ABM, Vancouver, Canada). The thermal cycling conditions were as follows: 95℃ for 3 min; followed by 40 cycles of 95 °C for 30 s and 60 °C for 10 s. The absolute quantification in each sample was performed by interpolating its Ct value into the linear regression equation derived from the standard curve, which correlated the Ct values with the logarithm of tachyzoite numbers [32].

### Statistical analysis

Data were presented as mean ± standard error of the mean (SEM) from at least three independent biological experiments. All statistical analyses were performed using GraphPad Prism 8.0 (GraphPad Software, San Diego, USA). The Shapiro–Wilk test confirmed that data for all groups showed no significant deviation from normality. Comparisons were made using a two-tailed Student’s *t*-test, one-way or two-way analysis of variance (ANOVA), or log-rank (Mantel–Cox) tests as indicated in the figure legends.

## Results

### *T. gondii* infection induces lung inflammation and injury

**Fig. 1:**
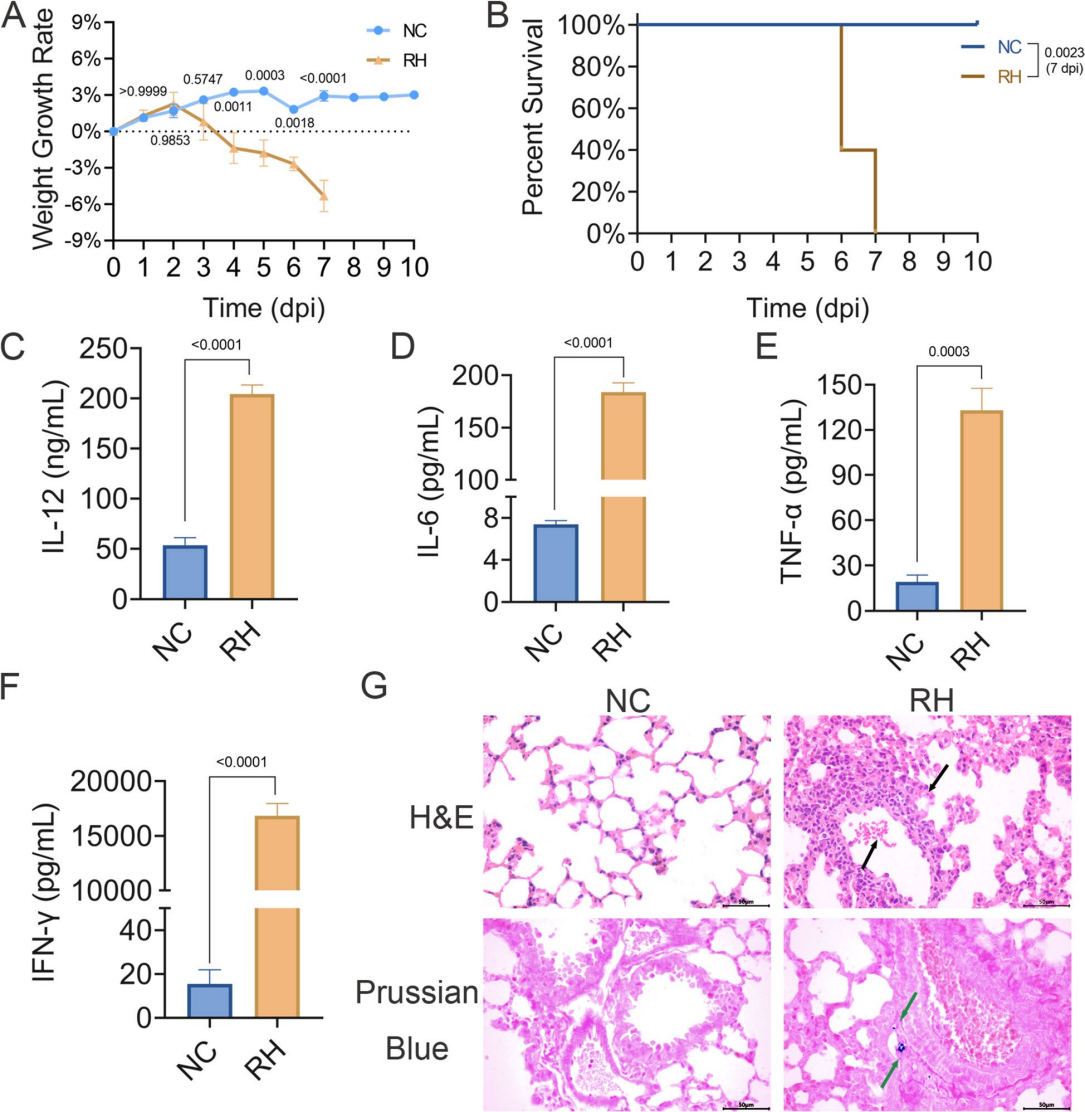
*Toxoplasma gondii* infection causes systemic inflammation, lung injury, and iron deposition in mice. Mice (*n* = 10/group) were injected i.p. with 1 × 10^3^ tachyzoites in the RH group, or an equal volume of PBS in the NC group. (**A)** Weight changes were recorded daily for 10 days. (**B)** Survival of mice was monitored for 10 days. The proinflammatory cytokines IL-12 (**C)**, IL-6 (**D)**, TNF-α (**E)**, and IFN-γ (**F)** were measured by ELISA. (**G)** Representative H&E and Prussian blue staining of lung sections from mice. Black arrows indicate alveolar hemorrhage and alveolar wall thickening; and green arrows indicate denote iron deposition (blue granules). Scale bar = 50 μm. Data are presented as mean ± standard error of the mean (SEM) of three independent experiments. Statistical significance was determined by two-way ANOVA with Šidák’s multiple comparisons test (**A**), log-rank test (**B**), or unpaired two-tailed Student’s *t*-test (**C**-**F**)

Lung injury is primarily characterized by thickening of alveolar septa and hemorrhage in alveolar spaces, accompanied by elevated inflammatory cytokines. To study *T. gondii*-induced lung injury, a mouse model was established. Mice were infected with *T. gondii* and observed daily. Beginning at 4 dpi, *T. gondii*-infected mice began to exhibit signs of illness, including ruffled fur, ascites, respiratory failure, and significant body weight loss (4 dpi, *P* = 0.0011; 7 dpi, *P* < 0.0001; Fig. 1A). Lethality was observed from 6 dpi, and all infected mice exhibited severe wasting and dyspnea by 7 dpi, with 100% mortality (*P* = 0.0023; Fig. 1B).

The proinflammatory cytokines levels of the infected mice were markedly elevated, including IL-12 (204.3 ± 9.0 pg/mL; *P* < 0.0001), IL-6 (183.9 ± 8.9 pg/mL; *P* < 0.0001), TNF-α (133.1 ± 14.5 pg/mL; *P* = 0.0003), and IFN-γ (16,816.9 ± 1134.7 pg/mL; *P* < 0.0001), indicating a strong systemic inflammatory response (Fig. 1C–F). In addition, lymphocytic infiltration, irregularly sized alveolar cavities, and pronounced interstitial congestion were observed in the lungs of *T. gondii*-infected mice by H&E staining (Fig. 1G). Moreover, significant iron accumulation in the pulmonary interstitium was detected by Prussian blue staining (Fig. 1G). These collected findings indicated the successful establishment of a lethal *T. gondii* infection model, with a heightened inflammatory cytokine response, lung pathology, and elevated iron accumulation, which potentially linked to ferroptosis.

### *T. gondii* infection triggers ferroptosis in mouse lungs

**Fig. 2:**
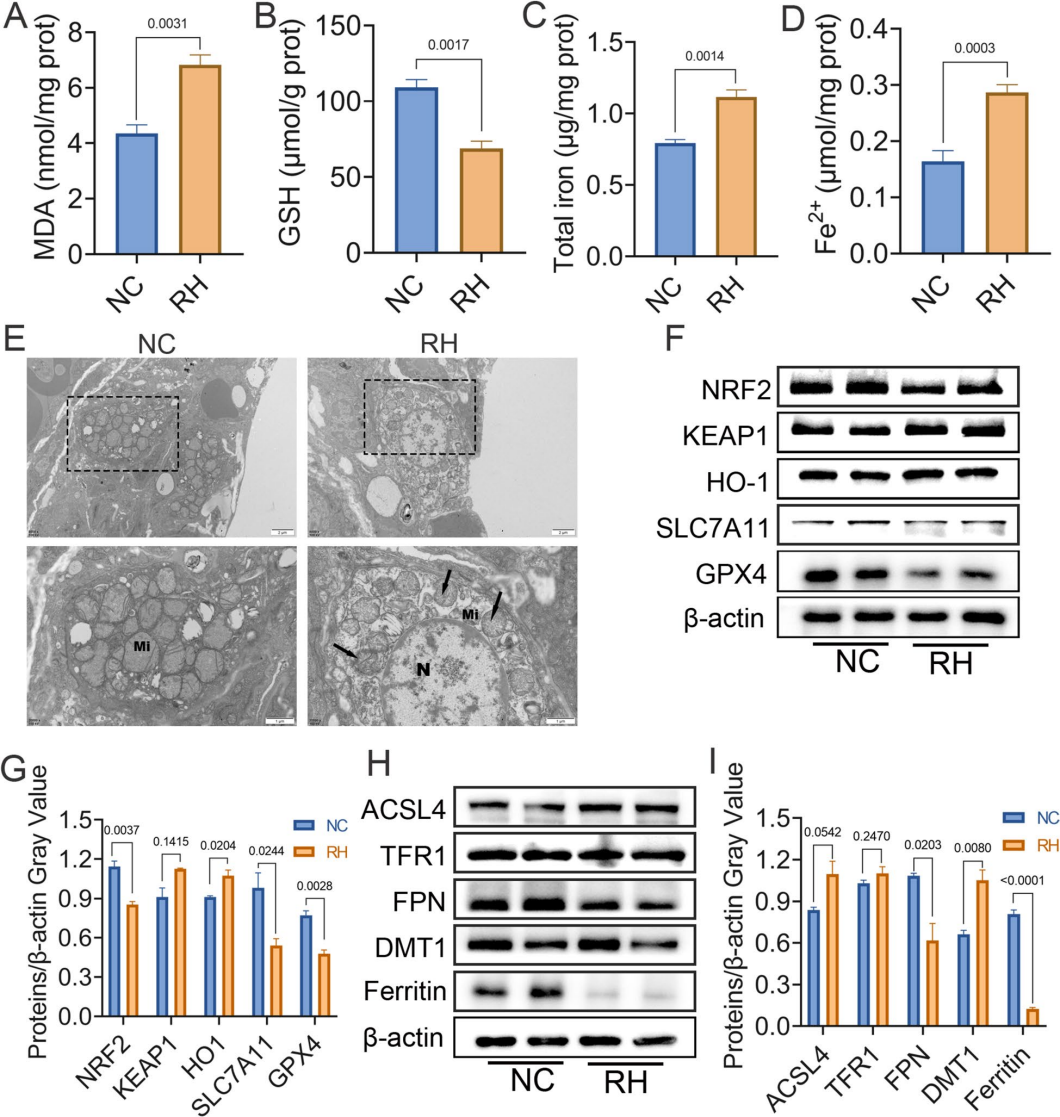
*Toxoplasma gondii* infection triggers ferroptosis in mouse lungs. Levels of MDA (**A**), GSH (**B**), total iron (**C**), and Fe^2+^ (**D**) in mouse lung tissues. (**E**) Representative TEM images of mitochondrial ultrastructure in mouse lungs. Top: low-magnification overview (scale bar = 2 μm). Bottom: high-magnification view of boxed region (N, nucleus; Mi, mitochondria; black arrows point to mitochondrial shrinkage and cristae rupture. Scale bar = 1 μm. (**F**) Western blot analysis of NRF2, KEAP1, HO-1, SLC7A11, GPX4, and β-actin. (**G**) Relative gray values of proteins in (**F**) normalized to β-actin by ImageJ. (**H**) Western blot analysis of ACSL4, TFR1, FPN, DMT1, Ferritin, and β-actin. (**I**) Relative gray values of proteins in (**H**) normalized to β-actin by ImageJ. Data are presented as mean ± SEM of three independent experiments. Statistical significance was determined by unpaired two-tailed Student’s *t*-test

To investigate whether ferroptosis is involved in *T. gondii*-induced lung injury, we assessed multiple ferroptosis-associated markers. The results demonstrated that MDA levels, a marker of lipid peroxidation, were significantly elevated in the lungs of infected mice (6.8 ± 0.4 nmol/mg protein (prot); *P* = 0.0031; Fig. 2A), whereas GSH level was markedly decreased (68.8 ± 4.9 μmol/g prot; *P* = 0.0017; Fig. 2B), when compared with the NC group. In addition, both total iron (1.1 ± 0.1 μg/mg prot; *P* = 0.014; Fig. 2C) and Fe^2+^ concentrations (0.3 ± 0.01 μmol/mg prot; *P* = 0.0003; Fig. 2D) were significantly increased following *T. gondii* infection. TEM revealed normal mitochondrial morphology of lung cells in the NC group, while the mitochondria in the lung cells of infected mice appeared shrunken and had lost their cristae (Fig. 2E), indicating that the structure of mitochondria was disrupted by *T. gondii* infection.

Additionally, the expression of key ferroptosis-related proteins involved in antioxidant, iron metabolism, and lipid metabolism pathways were analyzed in lung tissues by western blotting. The expressions of GPX4 (*P* = 0.0028), SLC7A11 (*P* = 0.0244), and NRF2 (*P* = 0.0037) were significantly decreased, whereas HO-1 expression was upregulated (*P* = 0.0204) (Fig. 2F, G). In the iron metabolism pathway, expression of FPN (*P* = 0.0203) and ferritin (*P* < 0.0001) was significantly reduced, while DMT1 expression was increased (*P* = 0.0080) (Fig. 2H, I). The lipid-peroxidation driver ACSL4, along with KEAP1 and TFR1, showed upward trends, though these changes were not statistically significant. These results suggested that *T. gondii* infection may trigger ferroptosis in mouse lungs by activating iron metabolism pathways while suppressing antioxidant pathways.

### Deferiprone attenuates lung ferroptosis in *T. gondii*-infected mice

**Fig. 3:**
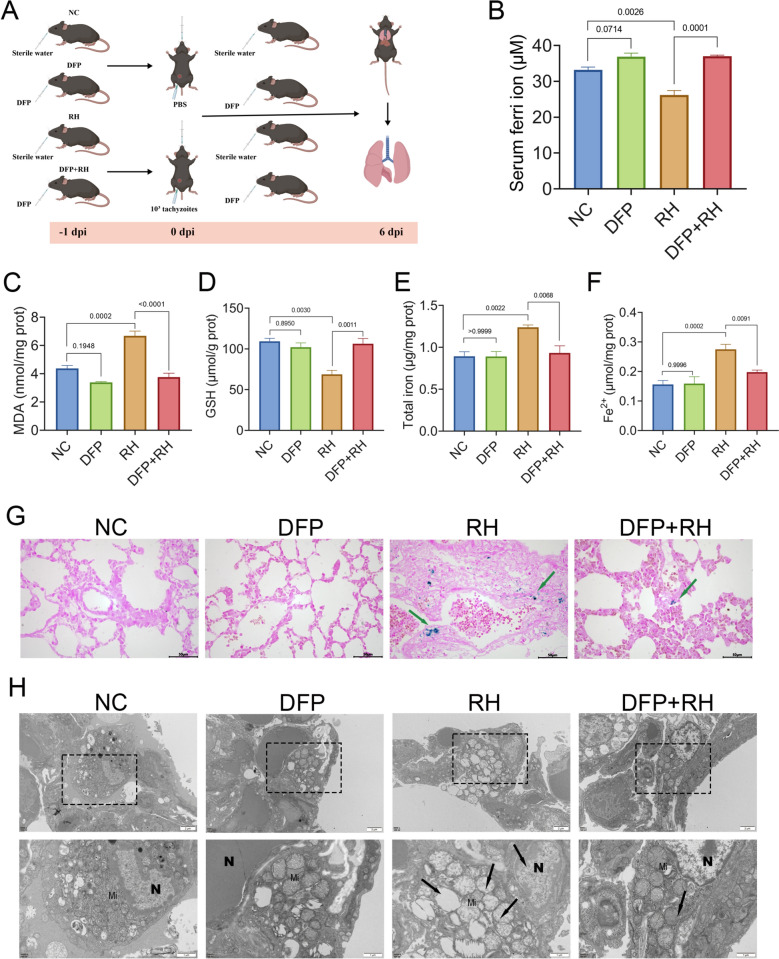
Deferiprone reduces *T. gondii*-induced oxidative stress, iron overload, and mitochondrial damage in mouse lungs. (**A)** Schematic of the experimental design for *T. gondii* infection and DFP treatment. From −1 to 6 dpi, mice in the NC and RH groups received water (i.g.), whereas the DFP and DFP + RH group mice received DFP (100 mg/kg, i.g.); mice in the RH and DFP + RH groups were injected i.p. with 1 × 10^3^ tachyzoites; *n* = 5 mice per group. (**B)** Serum iron concentration. The levels of MDA (**C)**, GSH (**D)**, total iron (**E)**, and Fe^2+^ (**F)** in lungs of mice. (**G)** Prussian blue staining of lung sections from mice at 6 dpi. Green arrows denote iron deposition (blue granules). Scale bar = 50 μm. (**H)** Representative TEM images of mitochondrial ultrastructures in lungs. Top: low-magnification overview (scale bar = 2 μm). Bottom: high-magnification view of boxed region (N, nucleus; Mi, mitochondria; black arrows point to mitochondrial shrinkage and cristae rupture. Scale bar = 1 μm). Data are presented as mean ± SEM from three independent experiments. Statistical significance was determined by one-way ANOVA with Tukey’s multiple comparison test

**Fig. 4:**
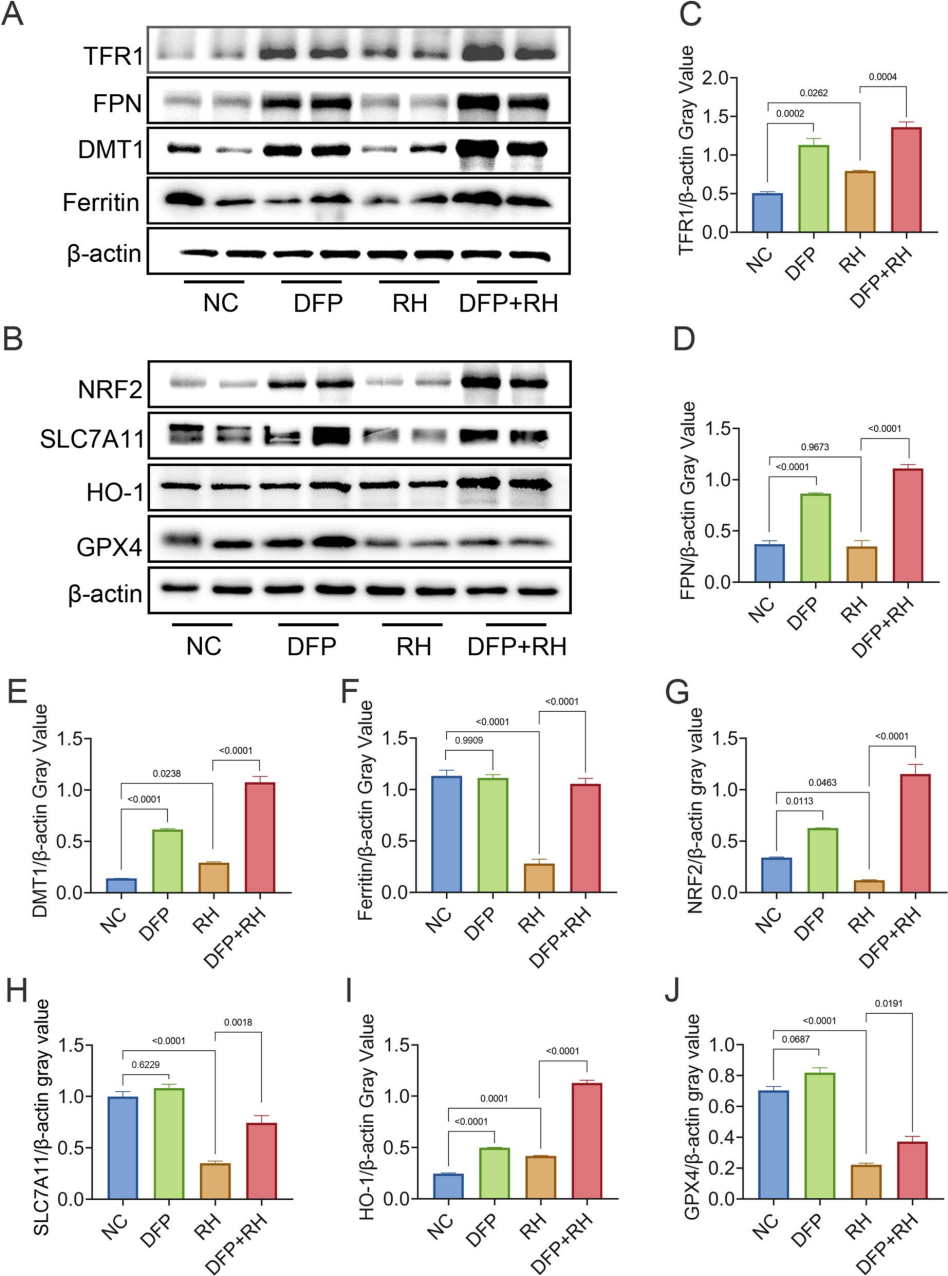
Antioxidant and iron metabolism pathways in *T. gondii-*induced lung ferroptosis were modulated by DFP. Western blot analysis of (**A**) antioxidant proteins (NRF2, SLC7A11, HO-1, and GPX4) and (**B**) iron metabolism proteins (TFR1, FPN, DMT1, ferritin) in mouse lung tissues. Relative gray values of (**C**) TFR1, (**D**) FPN, (**E**) DMT1, (**F**) ferritin, (**G**) NRF2, (**H**) SLC7A11, (**I**) HO-1, and (**J**) GPX4 expression were normalized to β-actin by ImageJ. Data are presented as mean ± SEM from three independent experiments. Statistical significance was determined by one-way ANOVA with Tukey’s multiple comparison test

To assess the effect of DFP on lung ferroptosis during *T. gondii* infection, we implemented the design outlined in Fig. 3A. The serum iron concentrations were significantly decreased (26.2 ± 1.2 μM; *P* = 0.0026) by *T. gondii* infection, but this decrease was greatly restored by DFP treatment (37.1 ± 0.3 μM; *P* = 0.0001; Fig. 4B). In addition, the trend of *T. gondii*-increased MDA was significantly reduced (3.8 ± 0.3 nmol/mg prot; *P* < 0.0001; Fig. 3C) after DFP treatment. The reduced GSH levels caused by *T. gondii* infection were substantially restored after DFP treatment (106.4 ± 6.3 μmol/g prot; *P* = 0.0011; Fig. 3D). Both total iron content, which elevated in the RH group (1.2 ± 0.03 μg/mg prot), and Fe^2+^ levels (0.3 ± 0.02 μmol/mg prot) were significantly decreased by DFP treatment to 0.9 ± 0.09 μg/mg prot (*P* = 0.0068; Fig. 3E) and 0.2 ± 0.007 μmol/mg prot (*P* = 0.0091; Fig. 3F)), respectively.

Prussian blue staining further confirmed that the iron accumulation was markedly reduced in the lungs of infected mice after DFP treatment (Fig. 3G). Moreover, the mitochondrial integrity in the lungs was significantly improved by DFP treatment, with TEM observation showing fewer deformed mitochondria and reduced cristae loss compared with the RH group (Fig. 3H). Taken together, these results suggested that DFP partially reversed the ferroptosis-associated alterations in the lungs of *T. gondii*-infected mice by reducing lipid peroxidation, restoring antioxidant capacity, ameliorating iron overload, and protecting mitochondrial structure. Administration of DFP alone did not cause the statistical change of these ferroptosis-related indicators.

### Deferiprone modulates antioxidant and iron metabolism pathways to inhibit lung ferroptosis in *T. gondii*-infected mice

To investigate the molecular mechanisms underlying *T. gondii*-induced ferroptosis, the key proteins involved in iron metabolism (Fig. 4A) and antioxidant (Fig. 4B) responses were analyzed after DFP treatment. The *T. gondii*-caused expressions of TFR1 (*P* = 0.0004; Fig. 4C), FPN (*P* < 0.0001; Fig. 4D), DMT1 (*P* < 0.0001; Fig. 4E), and ferritin (*P* < 0.0001; Fig. 4F) were significantly upregulated by DFP treatment. In parallel, the expressions of NRF2 (*P* < 0.0001; Fig. 4G), SLC7A11 (*P* = 0.0018; Fig. 4H), HO-1 (*P* < 0.0001; Fig. 4H), and GPX4 (*P* = 0.0191; Fig. 4J) were restored following DFP treatment. These findings indicated that *T. gondii* induced lung ferroptosis by targeting iron metabolism and antioxidant pathways, and this process was suppressed by DFP via chelation of excess iron in lung tissues.

The administration of DFP alone upregulated the expressions of molecules in the iron metabolism pathway, namely TFR1 (*P* = 0.0002; Fig. 4C), FPN (*P* < 0.0001; Fig. 4D), and DMT1 (*P* < 0.0001; Fig. 4E), and in the antioxidant pathway, namely NRF2 (*P* = 0.0113; Fig. 4G) and HO-1 (*P* < 0.0001; Fig. 4I).

**Fig. 5:**
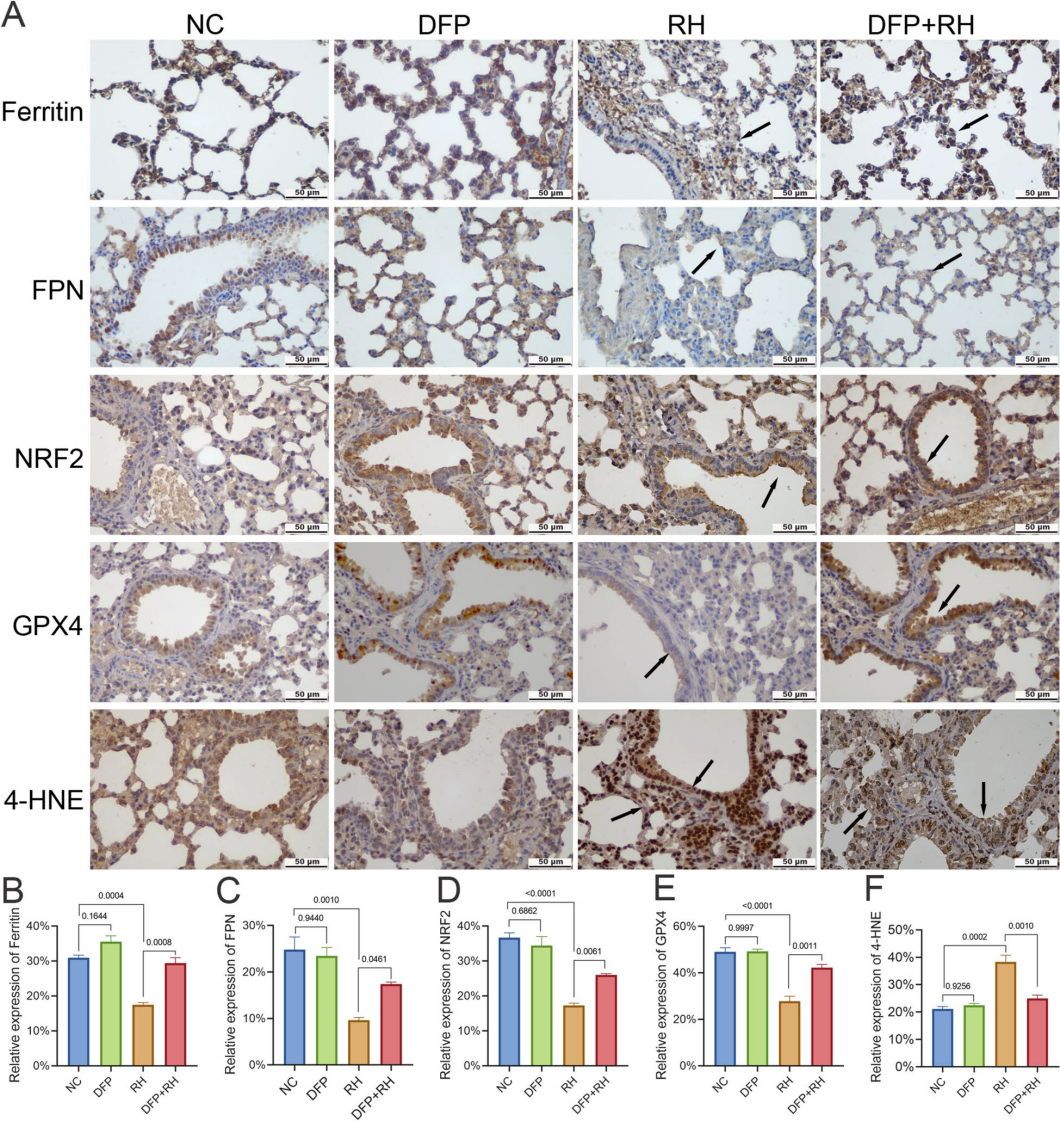
Immunohistochemical analysis of the key ferroptosis-related proteins in the lungs of *T. gondii*-infected mice. (**A**) Representative IHC staining of lung sections from mice. Black arrows indicate regions with differential protein expression. Scale bar = 50 μm. Semiquantitative analysis of the lung sections was performed to determine the relative expression of ferritin (**B**), FPN (**C**), NRF2 (**D**), GPX4 (**E**), and 4-HNE (**F**) by ImageJ. Statistical significance was determined by one-way ANOVA with Tukey’s multiple comparison test

Subsequently, to further confirm *T. gondii*-induced ferroptosis in lungs, ferroptosis key proteins of iron metabolism pathway (ferritin, FPN), antioxidant pathway (NRF2, GPX4) and lipid peroxidation products (4-HNE) were detected and observed by IHC analysis (Fig. 5A). Consistent with the western blot results, decreased levels of ferritin (*P* = 0.0004; Fig. 5B) and FPN (*P* = 0.0010; Fig. 5C) were observed in *T. gondii*-infected lungs. However, these changes were greatly reversed by DFP treatment, indicating the occurrence of iron metabolism pathway-mediated ferroptosis. In parallel, *T. gondii*-suppressed NRF2 (*P* = 0.0061; Fig. 5D) and GPX4 (*P* = 0.0011; Fig. 5E) expressions were greatly restored by DFP treatment, indicating that the activation of the antioxidant pathway also contributed to the alleviation of ferroptosis. Furthermore, 4-HNE (*P* = 0.0002; Fig. 5F), which is one of the key ferroptosis biomarkers, was strongly positive in both the nuclei and cytoplasma of *T. gondii*-infected mouse lungs and can be also inhibited by DFP (*P* = 0.0010; Fig. 5F). These collective data further confirmed that *T. gondii* can induce ferroptosis in the lungs by inhibiting iron metabolism pathway and antioxidant pathway. Administration of DFP alone did not cause statistical change of these ferroptosis-related indicators.

### Therapeutic efficacy of deferiprone in ameliorating *T. gondii*-induced lung injury

**Fig. 6:**
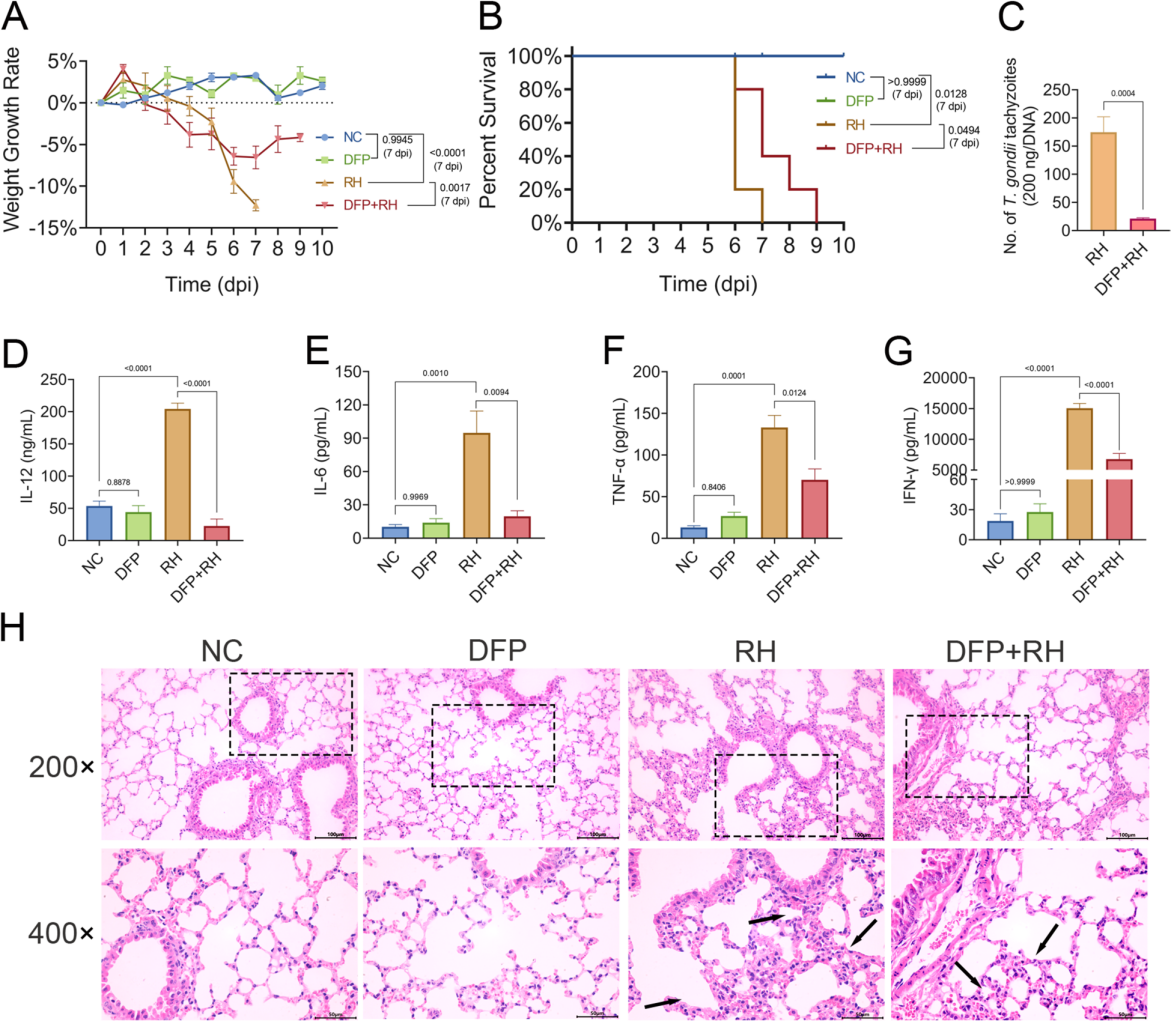
*Toxoplasma gondii*-induced mortality and lung pathology were mitigated by DFP treatment. (**A)** Body weight changes were recorded daily for 10 days. (**B)** Survival curves were monitored for 10 days. (**C)** Parasite burden in lungs from RH- and DFP + RH-treated mice were quantified by qPCR. The inflammatory cytokines IL-12 (**D)**, IL-6 (**E)**, TNF-α (**F)**, and IFN-γ (**G)** were measured by ELISA (*n* = 5 mice/group). (**H)** Representative H&E staining of lung sections from mice were performed. Black arrows indicate alveolar hemorrhage and alveolar wall thickening. Top: low-magnification (scale bar = 100 μm). Bottom: high-magnification (scale bar = 50 μm). Data are presented as mean ± SEM from three independent experiments. Statistical significance was determined by two-way ANOVA with Šidák’s multiple comparisons test (**A**), log-rank test (**B**), unpaired two-tailed Student’s *t*-test (**C**), or one-way ANOVA with Tukey’s multiple comparison test (**D**-**G**)

We investigated whether DFP attenuated *T. gondii*-induced lung injury. DFP treatment not only mitigated body weight loss at 7 dpi (*P* = 0.0017 versus RH group; Fig. 6A) but also significantly prolonged the survival time (*P* = 0.0494 versus RH group; Fig. 6B) and achieved a dramatic 72.6% reduction in lung parasite burden (*P* = 0.0004 versus RH group; Fig. 6C) in *T. gondii*-infected mice. In addition, the levels of IL-12 (45.1 ± 9.6 pg/mL; *P* < 0.0001), IL-6 (19.7 ± 5.0 pg/mL; *P* = 0.0094), TNF-α (70.2 ± 13.1 pg/mL; *P* < 0.0124), and IFN-γ (6756.3 ± 947.9 pg/mL;* P* < 0.0001) were markedly reduced in the DFP + RH groups compared with the RH group (Fig. 6D–G). H&E staining of lungs revealed that alveolar wall thickening was significantly mitigated, inflammatory cell infiltration was reduced, and alveolar collapse was prevented in the DFP + RH group (Fig. 6H). No significant differences in body weight, survival, cell cytokines, and lung pathological change were detected between the DFP and NC groups.

## Discussion

*T. gondii* infection could induce multiple programmed cell deaths, including host cell apoptosis, pyroptosis, autophagy, and necroptosis, which are involved in host inflammation, tissue injury, parasite clearance, and parasite survival [22]. *T. gondii* promotes apoptosis to reduce toxin accumulation in human leukemia T cell lines and induces pyroptosis, which mediates inflammatory storms that compromise tissue integrity [41–43]. In addition, *T. gondii* induces autophagy to eliminate intracellular parasites through IFN-γ and CD40/CD40L signaling [44]. Conversely, *T. gondii* secretes *TgNSM* to inhibit necroptosis, thereby promoting parasite survival [45]. Previous studies have shown that ferroptosis occurred in *T. gondii-*infected RAW264.7 macrophages and Vero cells, and eyes, spleen, liver, as well as kidney tissues of mice, marked by iron overload, lipid peroxidation, GSH and SLC7A11/GPX4 axis suppression, and ultrastructural mitochondrial damage [30, 32]. Similarly, in the present study, these indicators of ferroptosis were also detected in the lungs of *T. gondii*-infected mice. In addition, we also revealed dysfunction in the NRF/HO1 and pathway, decreased FPN and ferritin, as well as increased TFR1 and DMT1 in the iron metabolism pathway. These findings indicate that ferroptosis occur in the lungs of *T. gondii*-infected mice, which enrich the data of *T. gondii-*induced ferroptosis.

Lung injury, a common critical life-threatening syndrome, is driven in its pathogenesis by the key factor of inflammation. In bacterial, viral, and parasitic infections, mechanisms of lung injury include infection of lungs by pathogens, excessive inflammatory response, dysfunction or deficiency of surface-active substances, apoptosis and necrosis of lung cells, and oxidative stress in lung tissues, among other factors [46]. Currently, *T. gondii*-induced lung injury is characterized by excessive inflammatory cell activation, proinflammatory mediator production, pulmonary edema, and apoptosis [12, 47, 48]. Multiple pathogenic infections could activate ferroptosis to induce lung injury. For instance, SARS-CoV-2-triggered ferroptosis is associated with lung injury, characterized by hyaline membranes lining the alveolar walls or congestion and hemangiomatosis-like changes in the alveolar wall [49]. Secretion of tyrosine phosphatase A by *M. tuberculosis* exacerbates pathological lung damage and drives increased the load of *Mycobacteria* and acid-fast bacilli through the induction of ferroptosis [50]. Similarly, in the present study, the inhibition of ferroptosis induced by *T. gondii* significantly mitigated alveolar wall thickening, inflammatory cell infiltration, and alveolar collapse, indicating *T. gondii*-induced ferroptosis contributes to lung injury, which promotes understanding of the pathogenesis of intracellular parasitic infections.

Ferroptosis is primarily driven by iron metabolism, antioxidant, and lipid metabolism pathways [16]. *Salmonella* infection activates iron metabolism pathway to induce ferroptosis, marked by hepcidin-mediated FPN degradation, leading to hypoferremia, and inhibition of this pathway reduces bacterial load and improves survival rates in mice [25, 51]. In our study, *T. gondii*-infected mice also exhibited downregulated ferritin and FPN expression, and upregulated DMT1 expression, culminating in iron overload that induced ferroptosis. *Pseudomonas aeruginosa* exploits host polyunsaturated phosphatidylethanolamines to inducing ferroptosis in bronchial epithelium [52]. Both *M. tuberculosis* and SARS-CoV-2 induce excess ROS production, driving ferroptosis through the disruption of antioxidant pathways [25, 26, 52]. In the present study, we demonstrated that antioxidant pathways, including NRF2 and SLC7A11 downregulation, and GSH depletion, were also activated in *T. gondii-*induced ferroptosis. SLC7A11 is a component of system Xc⁻, which imports cystine, the precursor for GSH synthesis. GSH, the body’s principal antioxidant, is an essential cofactor for GPX4 activity [17, 53]. GPX4 utilizes GSH to reduce cytotoxic lipid hydroperoxides into non-toxic lipid alcohols, thereby preventing membrane damage and cell death [54–56]. However, lipid metabolism pathways-related proteins were not activated in *T. gondii-*infected lungs. These findings demonstrate that iron metabolism and antioxidant pathway are key pathways in *T. gondii-*induced lung ferroptosis.

Recent studies have developed various ferroptosis inhibitors targeting the iron metabolism pathway, the lipid metabolism pathway, and the antioxidant action [57, 58]. In our study, iron metabolism pathway was found involved in *T. gondii*-induced ferroptosis. DFP, an oral iron chelator approved by both the US Food and Drug Administration and the European Medicines Agency, is used worldwide for the treatment of iron overload [49]. DFP has demonstrated therapeutic efficacy in thalassemia, Alzheimer’s disease, Parkinson’s disease, SARS-CoV-2, *P. berghei*, and *M. tuberculosis* [23, 49, 59, 60]. A previous study suggested that DFP reduced the iron uptake and ameliorated *Toxoplasma*-induced retinochoroiditis by reducing retinal inflammation [30]. Another study showed that DFP led to ferroptosis resistance, attenuated pathological changes and inflammatory reactions, and reduced the *T. gondii* burden of the cerebral toxoplasmosis mouse model using TgCtwh3 [31]. In our study, the DFP administration markedly inhibited *T. gondii*-induced ferroptosis, as evidenced by decreased lipid peroxidation and iron overload, upregulated the expression of ferritin and FPN, and increased the activity of TFR1 and DMT1. In the antioxidant pathway, DFP treatment increased the expression of SLC7A11, GPX4, NRF2, and HO-1. Concurrently, DFP treatment prolonged the survival of mice during *T. gondii* acute infection, reduced the lung parasite burden, alleviated systemic inflammation, and attenuated lung injury. These findings are consistent with previous reports demonstrating the protective effects of iron chelators in *T. gondii*-associated encephalitis and retinochoroiditis [28, 31, 58], and our research further supplements the role of DFP in *T. gondii*-induced lung injury. Therefore, DFP may represent a promising adjunct therapy for toxoplasmosis, particularly in cases involving pulmonary complications or iron dysregulation.

## Conclusions

Our study revealed that ferroptosis, triggered by compromised dysregulated iron metabolism and antioxidant defenses via lipid peroxidation, represented a critical pathway in *T. gondii*-induced lung injury. DFP, acting as an iron-sequestering ferroptosis inhibitor, mitigated this damage through iron metabolic reprogramming. This work unveils a new pathological axis in toxoplasmosis and proposes ferroptosis-targeted interventions as a viable treatment paradigm.

## Supplementary Information

Additional file 1.

## Data Availability

The datasets supporting the findings of this article are included within the paper.

## Abbreviations

PBS: Phosphate-buffered saline
PBST: Phosphate-buffered saline with Tween 20
IL-12: Interleukin 12
IL-6: Interleukin 6
IFN-γ: Interferon γ
TNF-α: Tumor necrosis factor α
ELISA: Enzyme-linked immunosorbent assay
prot: Protein
PVDF: Polyvinylidene fluoride
SDS–PAGE: Sodium dodecyl sulfate–polyacrylamide gel electrophoresis
