# Supplementary material for: Toxoplasma gondii-induced ferroptosis contributes to acute lung injury in mice
Source: Parasit Vectors. 2025 Dec 28;19:55. doi: 10.1186/s13071-025-07207-x (PMC12859960; doi:10.1186/s13071-025-07207-x)
Supplement: Supplementary file 1 — Additional file 1. [file 13071_2025_7207_MOESM1_ESM.docx]

***Toxoplasma gondii*-induced ferroptosis contributes to acute lung injury in mice**

**Xiaodan Yuan, Zhenzhen Liu, Yeting Ma, Feixue Liu, Penglin Bao,** **Boya Du, Xu Zhang, Pengtao Gong, Nan Zhang, Jianhua Li, Xin Li^*^, Xiaocen Wang^*^**

State Key Laboratory for Zoonotic Diseases, Key Laboratory for Zoonosis Research of the Ministry of Education, Institute of Zoonosis, and College of Veterinary Medicine, Jilin University, Changchun 130062, China.

*Correspondence: E-mail addresses: Xiaocen Wang: wangxiaocen2016@163.com.

Tel: +86 43187836172, Fax: +864318798131; Xin Li: lixin2018@jlu.edu.cn.

E-mail addresses:

Xiaodan Yuan: yuanxiaodan0118@outlook.com

Zhenzhen Liu: zhenzhenliu@jlu.edu.cn

Yeting Ma: 18535444856@163.com

Feixue Liu: liufx23@mails.jlu.edu.cn

Penglin Bao: baopl21@mails.jlu.edu.cn

Boya Du: 13144305657@163.com

Xu Zhang: zhangxu1311@163.com

Pengtao Gong: gong_pengtao@126.com

Nan Zhang: zhangnangrace@163.com

Jianhua Li: jianhuali7207@163.com

Xin Li: lixin2018@jlu.edu.cn

Xiaocen Wang: wangxiaocen2016@163.com

**Table S1. Definition and implementation of humane endpoints and scoring sheets.**

| **Parameters** | **Score** |
| --- | --- |
| **Body weight** |  |
| 5–10% weight loss | 1 |
| 11–15% weight loss | 2 |
| 16–20% weight loss | 3 |
| >20% weight loss | Euthanasia |
| **Hair condition** |  |
| Slight piloerection | 2 |
| Prominent piloerection | 3 |
| Body temperature |  |
| Up to 2 °C above normal | 2 |
| Up to 3 °C above normal | 3 |
| Hypothermia (<35 °C) | Euthanasia |
| **Respiratory function** |  |
| Tachypnea | 2 |
| Dyspnea | 3 |
| **Pain parameters** |  |
| Moderately present orbital tightening | 1 |
| Intense present orbital tightening | Euthanasia |
| Eye discharge | 1 |
| Chest breathing | 2 |
| Arched posture | 3 |
| **Environment** |  |
| Soft stools or diarrhea | 1 |
| Bloody diarrhea | Euthanasia |
| **Behavior** |  |
| Tense or nervous during handling | 1 |
| In noticeable distress during handling such as tremors, vocalizations, and aggressiveness | 3 |

This table was adapted from Arruda et al. (2024) Animals (Basel), licensed under CC BY 4.0 [36].

**Table S2. Manners of death in *T. gondii*-infected mice.**

| **Group** | **Manner of Death** | **6 dpi** | **7 dpi** | **Total** |
| --- | --- | --- | --- | --- |
| RH | Found dead _a_ | 0/5 | 0/5 | 5 |
|  | Euthanized _b_ | 3/5 | 2/5 |  |

_a_ Mice were found dead spontaneously before the humane endpoint criteria were met.

_b_ Mice were euthanized upon reaching humane endpoint criteria.

n = 5 mice per group.

**Table S3.** **Manners of death in *T. gondii*-infected mice with or without DFP treatment.**

| **Group** | **Manner of death** | **6 dpi** | **7 dpi** | **8 dpi** | **9 dpi** | **Total** |
| --- | --- | --- | --- | --- | --- | --- |
| RH | Found dead _a_ | 0/5 | 0/5 | NP _c_ | NP _c_ | 5 |
|  | Euthanized _b_ | 4/5 | 1/5 | NP _c_ | NP _c_ |  |
| DFP+RH | Found dead _a_ | 0/5 | 0/5 | 0/5 | 1/5 | 5 |
|  | Euthanized _b_ | 1/5 | 2/5 | 1/5 | 0/5 |  |

_a_ Mice were found dead spontaneously before the humane endpoint criteria were met.

_b_ Mice were euthanized upon reaching humane endpoint criteria.

_c_ Not performed (NP) due to prior mortality.

n *=* 5 mice per group.
